# Reevaluation of reference values for bone marrow differential counts in 236 healthy bone marrow donors

**DOI:** 10.1007/s00277-020-04255-4

**Published:** 2020-09-15

**Authors:** Stefani Parmentier, Michael Kramer, Swetlana Weller, Ulrich Schuler, Rainer Ordemann, Gabi Rall, Markus Schaich, Martin Bornhäuser, Gerhard Ehninger, Frank Kroschinsky

**Affiliations:** 1 Department of Hematology, Oncology and Palliative Care, Rems-Murr-Hospital, Winnenden, Germany; 2 Medical Department I, University Hospital Dresden, Fetscherstr. 74, 01307 Dresden, Germany; 3 DKMS German Bone Marrow Donor Center, Tübingen, Germany

**Keywords:** Bone marrow, Cytology, Hematopoiesis, Normal differential counts

## Abstract

Despite the increasing role of molecular markers, differential counts and morphology of hematopoietic cells in the bone marrow (BM) remain essential diagnostic criteria in hematological diseases. However, the respective reference values for BM myelogram commonly used came from small series with limited numbers of healthy individuals. We evaluated the myelograms of 236 healthy individuals who underwent unrelated bone marrow donation. Health check-ups were performed 4 weeks prior to harvest. Samples for this study, taken from the first aspiration, were stained according to the standard *Pappenheim* method. Three experienced investigators assessed cellularity, megakaryopoiesis, and differential counts independently. The median donor age was 31 (range 18–51) years. Predonation tests did not reveal any relevant morbidity. Thirty-seven out of 42 hypocellular marrow samples were from younger donors up to 39 years. Content of megakaryocytes was normal in 210 specimens (89%). Gender and body mass index had significant impact on hematopoiesis, whereas age had not. The number of erythroblasts was higher (about 32%) and the proportion granulopoiesis slightly lower (about 50%) compared with previous studies. Differential counts showed also some differences with respect to individual maturation stages in these lines. Interrater comparisons showed greater reliability for the assignment of cells to the different hematopoietic cell lines than for single-cell diagnoses. This study largely confirms the results for cell counts in normal human bone marrow available from previous reports and provides some insights into factors that affect individual cell populations. It also reveals substantial variability among even experienced investigators in cytological diagnoses.

## Introduction

In 1944, E. E. Osgood and A. J. Seaman described a differential count of bone marrow (BM) aspirates from 12 healthy men, which since then has been cited as a reference in *Wintrobe’s Clinical Hematology* [1, 2]. Fifty-seven years later, Barbara Bain assessed the percentage of cells in freshly obtained, non-anticoagulated bone marrow smears of 50 healthy subjects [3]. The cohort included 30 men and 20 women, aged between 21 and 56 years, who were in good health and free of current infection and allergic conditions. The most recent results of normal myelograms from 140 healthy subjects were presented by Diem et al. in 2012 [4]. Until today, most laboratories rely on these normal BM values obtained from only limited numbers of healthy subjects.

More than 100 bone marrow harvests from healthy unrelated donors are performed every year at Dresden University Hospital. We gathered marrow squash slides from over 400 individuals of this cohort harvested over a 4-year period at our institution to investigate myelopoiesis.

Despite the huge increase in knowledge on molecular pathogenesis and the use of these findings in diagnostic algorithms, bone marrow examination remains the gold standard for diagnosing and monitoring in hematological diseases, and normal bone marrow counts remain the basis for understanding and proper evaluation of pathological bone marrow changes. The aim of our study was to re-evaluate the myelogram in a larger population of healthy individuals to confirm or improve the available evidence, and to make hematological diagnostics more reliable.

## Design and methods

### Donors and harvests

Over a 4-year period, we gathered BM slides from 445 healthy subjects who underwent unrelated bone marrow donation at our institution on behalf of the German Bone Marrow Donor Center (DKMS). For this study, we randomly selected marrow samples of 236 individuals out of this cohort to evaluate retrospectively health data, blood counts, and marrow hematopoiesis.

All volunteers underwent predonation health check-ups about 4 weeks prior to the harvest including health history, physical examination, and chest x-ray. Laboratory analyses included fully automated peripheral blood counts (Sysmex XE-2100, Sysmex Europe GmbH, Norderstedt, Germany), common chemistry tests for liver and renal function, C-reactive protein, and infectious disease markers (EBV, CMV, HSV1/2, HIV, Treponema pallidum, hepatitis A, B, C). Serum ferritin was used to assess the iron homeostasis based on gender and age-adapted normal ranges. One-hundred seventy-two donors (72%) underwent an autologous blood donation at the day of check-up.

Donors gave written informed consent for harvest and marrow examination. Both interventions were approved by the institutional review board (EK 240102007) of the Technical University of Dresden and procedures were in accordance with the Helsinki Declaration of 1975, as revised in 2008.

The donors had not received growth factors. Bone marrow harvests were performed under general anesthesia. The first 4 mL of aspirated marrow was taken for this study and mixed in a 20 mL syringe for anticoagulation with 1 mL Di-Na-EDTA 1.107% (AlleMan Pharma GmbH, final concentration of EDTA being approximately 0.22%).

### Selection of samples and medical record review

The aim in the selection of samples for this study was to ensure high slide quality and to have sufficient numbers for statistical analyses.

Medical records were reviewed and laboratory results were collected from the central laboratory server. At least weekly physical activities were categorized as “regularly.” Infectious disease markers and lifestyle information were compared with data of the German standard population published by the German federal health authorities [5–9].

### Slide preparation and bone marrow evaluation

Squash slides were prepared from the anticoagulated bone marrow specimens by experienced laboratory technicians within 2 h as recommended by the WHO [10]. May-Grunwald-Giemsa method (Pappenheim) was used for staining as previously described [11–13].

Three morphologists who are experienced in this field for many years performed bone marrow evaluation independently. Examination was based on well-established references [2, 11, 14–16]. Each investigator assessed cellularity and content of megakaryopoiesis in low power magnification (100-fold) using a Nikon Eclipse E600 Microscope. Forty to 60% of hematopoietic cells and the presence of one to three megakaryocytes per low-power field defined cellularity and megakaryopoiesis as normal, respectively. Furthermore, the investigators each performed a 200-cell differential cell count in higher magnification (1000-fold) for a total of 600 cells per sample, which exceeded the number of 500 cells recommended by the WHO [10]. Cell counts were performed in areas containing few bare nuclei; the cells were well-spread and not overlapping, found in clusters, or artifactually distorted because of the spreading artifact.

### Statistical analysis

We recorded the donors’ health data, lab results, and differential counts of the marrow evaluations in a Microsoft Access data bank and then finally analyzed with the statistic program R® version 3.5.3. [17]. If not otherwise indicated, we report medians and means with observed ranges. The effect of different factors on hematopoietic cell lines was tested by univariate analysis using the Mann-Whitney *U* test respective Kruskal-Wallis test in case of more than two groups. Categorical variables were compared using the *χ*^2^ test. A *p* value of less than 0.05 was considered significant. The two-sided 95% reference range for the physiological myelogram was estimated with sample quantile method number seven as described by Hyndman and Fan [18]. The interrater reliability was estimated using Krippendorff’s alpha for interval-scaled data [19]. A value of zero indicates perfect random disagreement, whereas a value of one indicates perfect agreement. Alpha can assume negative values when coders consistently agree to disagree, follow different coding instructions or having a conflicting understanding of them. In addition, we fitted unconditional linear mixed-effects models for all continuous variables with a random intercept for the rater and calculated the models’ intra-class-correlation-coefficients [20] to estimate the proportions of variance that are explained by the different investigators.

## Results

### Donor characteristics and predonation tests

Table 1 shows the demographic features of the study population. Median age was 31 (range 18–51) years. Preexisting health disorders did not influence suitability for donation. In some patients, the test results for clinical chemistry (liver enzymes, renal function, serum electrolytes) were slightly outside the normal range. These results had no clinical significance in any case. Neither there were donors with iron deficiency or overload.

**Table 1:** Demographic data of study population (*N* = 236)

Feature	nav[*n*]	*n*(%)
Male/female	236	165 (70)/71 (30)
Age cohorts	236	
< 30 years		112 (47)
30–39 years		71 (30)
≥ 40 years		53 (22)
Body mass index	235	
< 25 kg/m^2^		121 (51)
25–29 kg/m^2^		84 (36)
≥ 30 kg/m^2^		30 (13)
Smoking status	232	
Non-smoker		162 (70)
Smoker		70 (30)
Physical activity	93	
Regularly		57/93 (61)
Not regularly		36/93 (39)
Daily alcohol use	230	
Men		34 (21)
Females		3 (4)
Allergic diathesis in history	226	
Yes		60 (27)
No		166 (73)
Hormonal contraception	51	
Yes		13 (25)
No		38 (75)

*nav* number of available observations

The seroprevalences for herpes simplex virus and cytomegalovirus among the donors were less than in the German national standard population. For Epstein-Barr and varicella-zoster viruses, the frequency of infections was comparable with the general population. While the proportion of smokers was representative, the cohort included fewer individuals with daily use of alcoholic beverages and more subjects with regular physical activities (data not shown).

Table 2 summarizes the assessments of peripheral blood counts based on the institutional normal limits. Hemoglobin concentration, white blood cell, and platelet counts were within the defined ranges in the great majority of donors and detected deviations were minimal. In contrast, for mean corpuscular hemoglobin concentration (MCHC) and lymphocyte count, we detected out-of-limit values in 25% and 16% of the cases.

**Table 2:** Peripheral blood counts: institutional normal limits and outliers among individuals of the study population

Parameter	Institutional normal limits			Out of limits in this study			
	Units	Lower	Upper	nav	*n* (%)	Lowest	Highest
Hemoglobin	mmol/L						
Female		7.4	10.7	71	0 (0)	–	–
Male		8.6	12.1	165	0 (0)	–	–
Hematocrit							
Female		0.37	0.47	71	3 (4.0)	0.35	–
Male		0.40	0.54	164	1 (0.6)	0.39	–
MCH	fmol	1.70	2.10	103	5 (4.8)	1.56	–
MCHC	mmo/L						–
Female		20.0	23.0	43	1 (0.9)	19.7	–
Male		19.0	22.0	60	15 (25.0)	–	23.3
MVC	fL	80	96	235	10 (4.2)	79	100
White blood cells	X10^9/L	3.80	9.80	236	4 (1.7)	–	11.7
Neutrophils	X10^9/L	1.80	7.55	233	3 (1.3)	1.74	8.75
Eosinophils	X10^9/L	0	0.49	225	7 (3.1)	–	0.77
Basophils	X10^9/L	0	0.20	223	0 (0)	–	–
Monocytes	X10^9/L	0.20	1.00	229	4 (1.7)	0.12	–
Lymphocytes	X10^9/L	1.50	4.00	233	37 (15.9)	0.73	4.04
Platelets	X10^9/L	150	400	236	0 (0)	–	–

*nav* number of available observations

### Bone marrow evaluations

Cellularity was diagnosed as normal, reduced, or increased in 190 (80%), 42 (18%), and 4 (2%) cases, respectively. Thirty-seven out of 42 hypocellular marrow samples were from younger donors aged up to 39 years. A normal content of megakaryocytes was found in 210 specimen (89%), while hypoplasia or hyperplasia of megakaryopoiesis was seen in 8 (3%) and 18 (8%) of cases.

Table 3 shows the results of the differentiated cell counts in the bone marrow. The right part of this table contains findings from previous studies for comparison. Table 4 summarizes the *p* values for the relevance of different factors that possibly influence hematopoiesis. Gender and body mass index did significantly influence hematopoiesis, whereas age did not. Compared with women, men had significantly more erythropoiesis (*p* = 0.0003) resulting in lower GE and ME ratios, but less lymphopoiesis. Increased BMI was correlated with erythropoiesis and inversely related to the neutrophils and therefore affected the GE/ME ratio too. In smokers, neutrophilic granulopoiesis was significantly increased, and regular physical activity and blood donation lead to an expansion of erythropoiesis. In addition, blood donation led to reduced numbers of monocytes and lymphocytes. The intake of hormonal contraceptives was associated with lower numbers of mature neutrophils and an increase in lymphocytes in female donors.

**Table 3:** Results for normal bone marrow myelogram in the study cohort of 236 donors based on counting by three independent investigators (200 cells each, in total 600 cells). The right part of this table shows the results from previous reports for comparison

Lineage and maturation	This study				Wintrobe (2009)	Bain (1996)	Diem (2012)
	Range (%)	Reference range (%)	Median (%)	Mean (%)	Mean (%)	Mean (%)	Mean (%)
Erythroblasts (total)	8.5–56.5	15.8–46.2	32.0	31.7	25.6		23.0
Male	11.0–54.5	16.2–46.6	33.0	32.4		28.1	
Female	8.5–56.5	15.6–45.0	29.5	30.1		22.5	
Proerythroblast	0–5.0	0–3.0	0.5	0.6	0.6		<1.0
Basophilic	0–21.0	0.5–13.5	3.5	4.5	1.4		1.0
Polychromatophilic	3.0–47.0	7.8–34.5	20.0	20.2	21.6		2.0
Orthochromatic	0–21.0	0.5–16.5	5.5	6.3	2.0		19.0
Granulocytes (total)	24.5–72.5	34.8–66.3	49.5	49.9			
Male	26.0–70.5	35.5–64.5	49.5	49.7			
Female	24.5–72.5	34.1–67.4	50.0	50.5			
Neutrophils (total)					53.6		55.0
Myeloblast	0–8.5	0–5.0	1.5	1.6	0.9	1.4	2.0
Promyelocyte	0–8.5	0–5.5	1.0	1.3	3.3	7.8	2.0
Myelocyte	1.0–31.7	5.8–24.0	13.5	13.7	12.7	7.6	3.0
Metamyelocyte	0.5–18.0	1.0–12.0	4.5	5.0	15.9	4.1	9.0
Band	2.0–34.0	6.5–26.2	14.5	15.2	12.4	32.1(m), 37.4(f)	10.0
Segmented	1.0–27.5	2.5–19.7	9.5	9.8	7.4		29.0
Eosinophils (total)	0–18.0	0.5–7.0	2.5	3.0	3.1	3.5	4.0
Basophils (and mast cells)	0–3.0	0–1.5	0	0.3	<0.1	0.1	<1.0
GE-ratio	0.5–5.9	0.8–4.1	1.6	1.7			
Monocytes	0–8.5	0–6.0	2.0	2.2	0.3	1.3	<1.0
ME ratio	0.6–6.2	0.8–4.1	1.6	1.8	2.3		
Male	0.6–6.1	0.8–4.0	1.6	1.8		2.1	
Female	0.6–6.2	0.8–4.2	1.7	2.0		2.8	
Lymphocytes	1.0–38.5	5.5–23.2	13.2	13.6	16.2	13.1	13.0
Plasma cells	0–17.5	0–7.0	2.3	2.6	1.3	0.6	1.0

The calculation of GE (granulopoiesis/erythropoiesis) and ME (myelopoiesis/erythropoiesis) ratios included all stages of maturation in the individual lines: myeloblast to segmented granulocyte for G, plus monocytes for M, and proerythroblast to orthochromatic erythroblast for E, respectively. *m* male, *f* female

**Table 4:** Univariate evaluation of factors with possible impact on hematopoiesis

Lineage and maturation	Gender	Age	BMI	Smoking	Alcohol daily	Physical activity	Blood donation	Allergic diathesis	Hormonal contraception
Erythroblasts (total)	*0.0003*	0.2391	*0.0009*	0.0722	0.6211	*0.0328*	*0.0014*	0.1969	0.2864
Proerythroblast	0.8429	0.2192	0.3975	0.5901	0.1178	0.8847	0.5500	0.5964	0.6122
Basophilic	0.3023	0.5864	*0.0080*	*0.0489*	0.5169	*0.0491*	0.3877	0.1532	0.2394
Polychromatophilic	*0.0126*	0.2486	0.2394	0.1248	0.6251	0.1105	*0.0006*	0.6768	0.6759
Orthochromatic	*0.0086*	0.4648	*0.0243*	0.7806	0.6066	0.6028	0.7834	0.3118	0.0693
Granulocytes (total)	0.2028	0.8452	*< 0.0001*	*0.0086*	0.5027	0.0961	0.3296	*0.0425*	*0.0289*
Myeloblast	0.0537	0.5637	*0.0212*	0.1275	*0.0131*	0.6628	0.6389	0.8523	0.7451
Promyelocyte	0.1488	0.6953	0.5145	0.9388	0.1955	0.8655	0.7976	0.1217	0.1857
Myelocyte	0.6845	0.4012	0.7894	0.3826	0.5081	0.3786	0.5410	0.2343	0.7557
Metamyelocyte	0.0928	0.3953	*0.0350*	0.5650	0.5585	0.5555	0.2481	0.9606	0.7755
Band	0.7724	0.8982	0.0822	0.0874	0.0879	0.4039	0.2370	*0.0029*	0.4002
Segmented	*0.0063*	0.3104	*0.0001*	0.3505	0.3731	0.0996	0.3014	0.5780	*0.0134*
Eosinophils	0.3825	0.3641	0.3025	0.3256	0.6372	0.0833	0.5524	0.0526	0.1014
Basophils	0.1463	0.1433	0.4304	0.1522	0.4788	0.6771	0.5159	0.6520	0.0672
GE-ratio	*0.0060*	0.5151	*0.0017*	*0.0236*	0.5450	0.1341	*0.0398*	0.1484	0.2052
ME ratio	*0.0060*	0.4881	*0.0021*	*0.0225*	0.5274	0.1541	*0.0246*	0.1521	0.2588
Monocytes	0.9012	0.8026	0.6173	0.7332	0.3714	0.1992	*0.0010*	0.4785	1.000
Lymphocytes	*0.0006*	0.0971	0.4337	0.4595	0.3745	0.0929	*0.0031*	0.1257	*0.0205*
Plasma cells	*0.0257*	*0.0131*	0.3774	0.2670	0.2094	0.9923	0.8993	0.4711	0.9009

Significant test results (*p* < 0.05) are delighted in italics. Granulocytes (total) includes also eosinophils and basophils. For the definition of GE and ME ratio see legend to Table 3

### Interrater reliability

As shown in Table 5, the results differed substantially between the investigators. The highest values for reliability were achieved for the assignment of cells to the different hematopoietic cell lines and cellularity, while single-cell diagnoses varied in a higher degree. There was also a substantial impact of the investigator on the variability of counting, ranging from < 1% for eosinophils to 58% for basophilic erythroblasts.

**Table 5:** Impact of investigators on test results

Lineage and maturation	ICC	Krippend alpha
Cellularity	0.012	0.3822
Megakaryopoiesis	0.000	0.3270
Erythroblasts (total)	0.027	0.3483
Proythroblast	0.356	− 0.1292
Basophilic	0.582	− 0.1699
Polychromatophilic	0.311	0.0130
Orthochromatic	0.108	− 0.0300
Granulocytes (total)	0.011	0.3409
Myeloblast	0.233	0.0242
Promyelocyte	0.530	− 0.1691
Myelocyte	0.196	0.0823
Metamyelocyte	0.346	− 0.0881
Band	0.292	− 0.0093
Segmented	0.275	0.1271
Eosinophils	0.009	0.3410
Basophils	0.049	0.0179
Monocytes	0.412	− 0.0854
Lymphocytes	0.059	0.2528
Plasma cells	0.132	0.2070

*IC-intra class correlation

Krippend alpha-Krippendorff’s alpha

Krippendorff’s alpha characterizes interrater reliability. A value of zero indicates perfect random disagreement, whereas *a* value of 1.0 indicates perfect agreement. Alpha can be negative when investigators consistently disagree, use different coding instructions or having conflicting understanding of them

Intra-class-correlation (ICC) estimates the proportions of variance due to the investigators. A value of zero indicates no impact; a value of 1.0 indicates that the investigators only explain variances

## Discussion

Our study is the largest and most comprehensive examination of physiological hematopoiesis published so far. There are some differences with respect to the number of included individuals, and preparation and assessment of the marrow samples as well between the data reported by Wintrobe [1, 2], Bain [3], Diem [4], and this study (Table 3). The results were cited by Wintrobe based on aspirates from only 12 males, on whom sternal punctures were performed, a procedure that is largely abandoned today. The study by Diem included a higher number of subjects, but did provide neither gender-specific information nor reported the site of interventions.

The references described in the papers differ mainly in the erythroblastic line and with respect to the neutrophilic cell counts. The number of erythroblasts we found was higher than the other authors did. However, the higher proportion of erythropoietic cells in males compared with women had been reported previously. In contrast, the total amount of granulopoietic cells in our series was lower than in the other papers. A difference between the male and female cohort concerning the mature neutrophils as described by Bain we found only for the segmented cells. There were also slightly different results in both lines, erythropoietic and granulocytic, for the proportions of individual maturation stages. This might be attributable to the more difficult and in some extent subjective single-cell diagnoses, which corresponds to the results of the interrater reliability tests. However, the number of myeloblasts, which is probably one of the most important features for the diagnosis in myelodysplasias and leukemias, was not different to the previous reports.

Among the other factors with possible relevance to the hematopoiesis, the physical constitution had a stronger impact than age. The increased erythroblast counts in overweight and obese individuals may be a consequence of a higher need for oxygen supply. Pronounced erythropoiesis after blood donation and due to regular physical activities is not surprising, and the increase in neutrophilic cells in smokers is a known phenomenon. In contrast, the pathophysiology behind the shifts of neutrophils and lymphocytes caused by oral contraceptives remains unclear. The frequent use of alcoholic beverages induces dyspoetic changes mainly in the erythroblasts. However, we did not found numeric changes within the hematopoietic lines in our study. To find the majority of hypocellular marrow samples in younger donors was an unexpected result and the interpretation is difficult.

In summary, except for elaborated differences mentioned above, we could confirm in a larger cohort the bone marrow values reported by Bain, which many laboratories use as references.

Because disorders of the hematopoietic system mainly affect patients above the age of 60 years, the lack of individuals out of this population represents the major limitation in our study. Data on bone marrow values in elderly individuals are rare. Samples from patients above the age 50 years are actually subject to another examination. Furthermore, the number of available observations in the analyzed subgroups is too small to draw definite conclusions.

The study also shows that cytological findings are highly subjective and influenced by the investigators. In this series, their experience in cytological diagnostics ranged from 2 to 10 years. If digital diagnostic devices using artificial intelligence systems might be helpful not only in terms of efficacy but also in obtaining objective information is doubtful as even the today available blood analyzers are not able to make precise cell diagnosis in pathologic situations.

## Data Availability

The slides have been archived at Medical Department I of Dresden University Hospital and all data are available in electronic files. The statistical analyses can be presented as PDF file from statistic program R®.
